# *In Vivo* Visualization of Stromal Macrophages via label-free FLIM-based metabolite imaging

**DOI:** 10.1038/srep25086

**Published:** 2016-05-25

**Authors:** Joseph M. Szulczewski, David R. Inman, David Entenberg, Suzanne M. Ponik, Julio Aguirre-Ghiso, James Castracane, John Condeelis, Kevin W. Eliceiri, Patricia J. Keely

**Affiliations:** 1 Molecular and Cellular Pharmacology Graduate Program, UW-Madison, Madison, WI, USA; 2 Department of Cell and Regenerative Biology, School of Medicine and Public Health, UW-Madison, Madison, WI, USA; 3 Laboratory for Optical and Computational Instrumentation, UW-Madison, Madison, WI, USA; 4 Gruss-Lipper Biophotonics Center, Albert Einstein College of Medicine, New York, NY, USA; 5 Department of Medicine, Mount Sinai, New York NY, USA; 6 College of Nanoscale Science and Engineering, SUNY Polytechnic University, Albany, NY, USA.

## Abstract

Macrophage infiltration and recruitment in breast tumors has been correlated with poor prognosis in breast cancer patients and has been linked to tumor cell dissemination. Much of our understanding comes from animal models in which macrophages are labeled by expression of an extrinsic fluorophore. However, conventional extrinsic fluorescence labeling approaches are not readily applied to human tissue and clinical use. We report a novel strategy that exploits endogenous fluorescence from the metabolic co-factors NADH and FAD with quantitation from Fluorescence Lifetime Imaging Microscopy (FLIM) as a means to non-invasively identify tumor-associated macrophages in the intact mammary tumor microenvironment. Macrophages were FAD^HI^ and demonstrated a glycolytic-like NADH-FLIM signature that was readily separated from the intrinsic fluorescence signature of tumor cells. This non-invasive quantitative technique provides a unique ability to discern specific cell types based upon their metabolic signatures without the use of exogenous fluorescent labels. Not only does this provide high resolution temporal and spatial views of macrophages in live animal breast cancer models, this approach can be extended to other animal disease models where macrophages are implicated and has potential for clinical applications.

## Macrophages and Cancer

Significant evidence in the literature suggests that stromal cell populations play a prominent role in the development and progression of cancer *in vivo*. In particular, macrophages recruited to the tumor significantly influence the extent of metastasis^1^,^2^,^3^,^4^,^5^,^6^,^7^,^8^,^9^, and recent findings demonstrate their critical role in tumor cell intravasation^10^. Consistent with this finding, genetic ablation of colony stimulating factor -1 (CSF-1), a cytokine that recruits macrophages, results in a decrease in macrophage recruitment to the breast tumor microenvironment, associated decreases in tumor blood vessel density and the formation of lung metastasis in the PyVT animal model^5^. Recent clinical data from a number of human cancers has correlated macrophage presence with a poorer patient prognosis^11^,^12^,^13^. Taken together these data suggest that macrophages play a prominent role in the progression of cancer. A greater understanding of tumor-associated macrophages will open new opportunities for better diagnosis, prognostic assessment, and targeted therapeutic approaches.

There is a critical need to be able to identify and image macrophages in live tissue or fresh biopsies. To date there have not been approaches that could specifically identify these macrophage populations in live tissue without the use of antibody labeling, exogenous dyes or genetic manipulations, such as the expression of EGFP. We outline here a novel imaging approach that can identify live, unstained macrophages from the tumor microenvironment *in vivo,* which exploits the endogenous cellular fluorescent signal without exogenous fluorophores or dyes.

### Endogenous Fluorescence to determine metabolic signatures

Endogenous fluorescence has been used as a source of contrast to image and characterize cell phenotype without the use of exogenous labels and dyes. Two major endogenous fluorophores in the tumor microenvironment are the intermediate metabolites nicotinamide adenine dinucleotide (NADH and NADPH) and flavin adenine dinucleotide (FAD). For NADH and NADPH, only the reduced forms are fluorescent (*e.g.* NADH, but not NAD+), and for FAD only the oxidized form is fluorescent (FAD but not FADH_2_). Both NADH and FAD are primary metabolic cofactors involved in glycolysis and cellular respiration, where their reduction and oxidation are prominent in the process of generating ATP. As the relative concentrations of the reduced and oxidized forms of these metabolic cofactors change with different levels of cellular metabolism, quantifiable NADH and FAD intensity imaging can be used to distinguish the metabolic state of a cell^14^,^15^,^16^. This technique has been exploited in predominantly *in vitro* studies to quantify the metabolic shifts observed in stem cell differentiation^14^ as well as changes in cancer cell biology^16^,^17^,^18^.

In addition to imaging the intensity of NADH, Fluorescence Lifetime Imaging Microscopy of NADH (NADH-FLIM) can provide information on the state of the cellular metabolic environment^19^. By utilizing time-correlated single photon counting (TCSPC), a histogram of photon counts vs. time from excitation can be created, which illustrates a fluorescence decay curve at each pixel^20^. These decay curves are then fitted to a bi-exponential decay to account for the two populations of NADH: a long lifetime representative of NADH bound to proteins and a short lifetime representative of free NADH^19^,^21^ By looking at both the weighted average fluorescence lifetime and the fractional contributions of the bound and free components, we can quantify changes in the relative concentrations of bound to free NADH.

Recently, it has been suggested that NADH-FLIM and its subsequent changes in the fractional contribution of free vs. bound NADH correlates with shifts in metabolism between aerobic glycolysis (expected to result in more free NADH in the cytosol) and oxidative phosphorylation (more bound NADH, especially in the mitochondria)^19^,^22^,^23^. Here, using Multiphoton Laser Scanning Microscopy (MPLSM) along with the use of a Mammary Imaging Window (MIW)^24^,^25^, we exploit intrinsic fluorescence intensity and FLIM imaging of FAD and NADH to characterize cellular metabolism, and as a novel way to identify distinct cell types within the breast tumor microenvironment *in vivo*. We find that stromal macrophages are readily distinguishable from tumor cells by their high FAD intensity and a highly glycolytic NADH-FLIM signature.

## Results

### Endogenous Fluorescence identifies subpopulations of stromal cells in the breast tumor microenvironment

**Figure 1:**
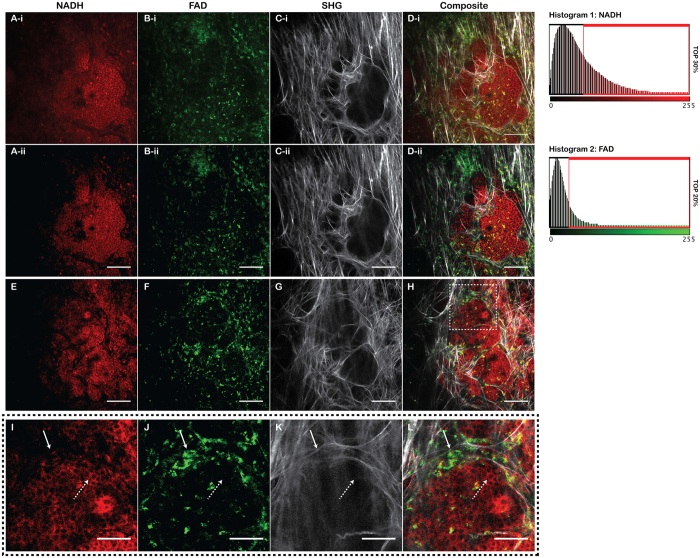
Endogenous Fluorescence shows significant contrast in the breast tumor microenvironment. Multiphoton laser scanning microscopy (MPLSM) of a PyVT tumor viewed through a mammary imaging window (MIW), taken during 2 independent imaging sessions. (**A–D**) are images from one animal (**E–H**) are from another experiment. (**I–L**) are enlarged view of the region boxed in from (**E–H**). (Ai, Bi, Ci, Di) are images showing the entire dynamic range collected from an imaging session. (Aii, Bii, Cii, Dii) are the same data but NADH^HI^ and FAD^HI^ after a threshold mask has been applied. NADH^HI^ cells are define as the top 30% of pixels with the greatest intensity. Histogram 1 shows pixel intensities levels from (Ai). The Red box shows the pixel values that are displayed in (Aii). FAD^HI^ cells are defined as the 20% pixels with greatest intensities. Histogram 2 shows pixel values that are displayed in (Bi). The Red box shows pixel values that are shown in (Bii). (Ai, Aii, E, I) Endogenous fluorescence from NADH, collected with a 445/20 BP filter at 780 nm 2-photon excitation. Scale Bar = 100 μm. (Bi, Bii, F, J) Endogenous fluorescence of FAD collected with a 562/30 BP filter at 890 nm 2-photon excitation. Scale Bar = 100 μm. (Ci, Cii, G, K) SHG images were collected with a 445/20 BP filter at 890 nm 2-photon excitation. Scale Bar = 100 μm. (Di, Dii, H, L) Composite image of images. Scale bar = 100 μm in (**A**–**H**,**I**–**L**) Enlarged view from (**E–H**). Scale bar = 20 μm. Solid arrow indicates an FAD^HI^ cell. Dashed arrow indicates an NADH^HI^ cell.

Building on previous studies from our group and others showing that the fluorescence of NADH and FAD can be used to image cells, we aimed to investigate if these endogenous fluorophores could be exploited to image specific subpopulations of stromal cells surrounding tumors. Imaging MMTV-PyVT mammary tumors through a MIW^24^ with multi-photon excitation microscopy, allowed us to observe tumor cell and surrounding stroma *in vivo*. (Fig. 1A). Tumor cells exhibited a high autofluorescent NADH signal intensity (Fig. 1Ai). NADH^HI^ cells represent the brightest 30% pixels, defined by the auto-threshold function of ImageJ (Fig. 1 Histogram 1, Fig. 1Aii). The tumor cells were surrounded by stromal collagen, which was imaged by second harmonic generation, SHG, (Fig. 1C,G), consistent with our previous *ex vivo* observations^26^.

In the stroma, a unique cell population was observed based on endogenous fluorescence: cells high in FAD intensity (FAD^HI^) (Fig. 1Bi), which were the brightest 20% pixels based on the ImageJ auto-threshold (Fig. 1 Histogram, Fig. 1Bii). FAD^HI^ cells were found predominantly outside of the tumor and concentrated mainly in the stroma, with a small population of FAD^HI^ cells also found inside of the tumor (Fig. 1Bi,Bii). Notably, the FAD^HI^ cells that reside inside of the tumor are often found along collagen fibers, consistent with findings that immune or stromal cells track along collagen to engage tumor cells^2^. Occasionally, cells were observed that were both FAD^HI^ and NADH^HI^, but these cells were not characterized further in this study. Thus, these results demonstrate at least two different sets of abundant cell types that can be distinguished by their endogenous fluorescence intensity: NADH^HI^ tumor cells and FAD^HI^ stromal cells. We consistently saw these populations across every imaging session, such as the example shown in Fig. 1E–H. A zoomed image of this field of view is shown in Fig. 1J–L.

### FAD^HI^ cells have macrophage markers

**Figure 2:**
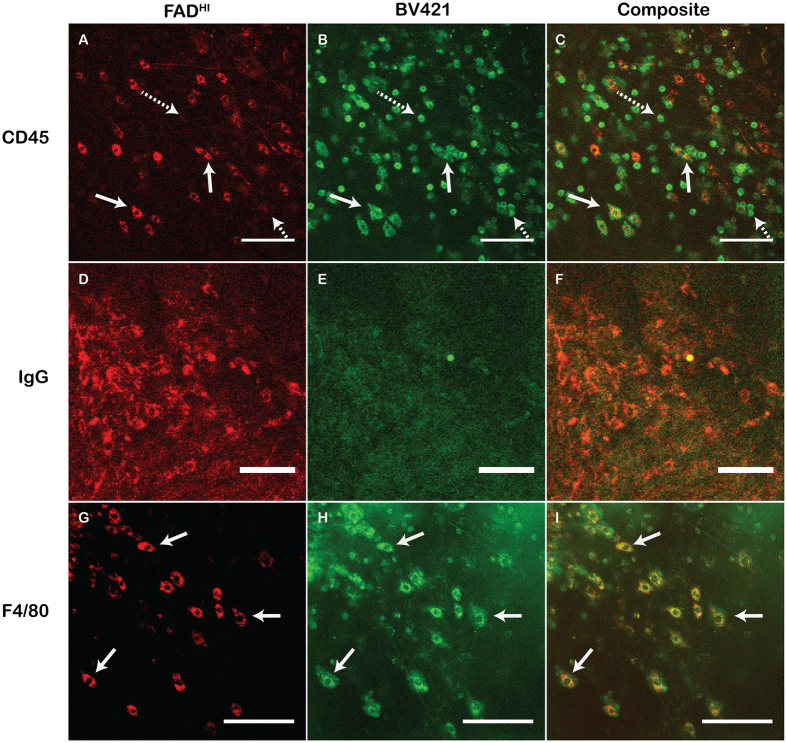
FAD^HI^ cells have macrophage markers. 2-MPLSM imaging of a PyVT tumor through a ported MIW. (**A**) Intensity image of FAD showing FAD^HI^ cells. (**B**) Visualization of BV421-conjugated CD45 antibody 5 min after being injected under the ported MIW. Solid arrows show FAD^HI^ cells that co-localize to CD-45 positive cells. Dashed arrows show cells that are CD45 positive only. **(C**) Composite of A and B. Scale bar A-C  = 50 um. (**D**) FAD^HI^ cells in PyVT tumor. (**E**) Visualization of BV421 conjugated to non-specific IgG antibody 20 min after injection under the ported MIW. **(F**) Composite of D and E. Scale bar D-F = 50 μm. (**G**) FAD^HI^ cells in a PyVT tumor under a MIW. **(H**) Visualization of BV421-conjugated F4/80 antibody. Arrows indicate cells that are FAD^HI^ and BV421-F4/80 positive. (**I**) Composite image of G and H. Scale bar G-I = 50 μm.

To identify the FAD^HI^ population of cells, we used a primary fluorescence-conjugated antibody that is specific for lymphocytes, Brilliant Violet 421-CD45. In order to inject primary conjugated antibodies under our MIW, we developed a ported MIW. This was created by inserting a 30 gauge hypodermic needle through a hole bored into the MIW frame. The needle was positioned such that it’s tip was located within the mammary tumor and imaging range of the microscope. Using a ported MIW, we were able to inject Brilliant Violet 421-CD45 antibody into a localized region under the imaging window into the tumor microenvironment. Several cells were both CD45^+^ and FAD^HI^ (Fig. 2A–C). However, only 55% of CD45^+^ were also FAD^HI^ (arrows in Fig. 2A–C). As CD45 labels all lymphocytes, these findings suggest that the FAD^HI^ cells represent just a subset of CD45^+^ cells. (Interestingly, some of the CD45^+^ cells that are not FAD^HI^ have a rounded morphology, which may represent T-lymphocytes.) As a control, BV421-IgG antibody was used, which did not specifically label any cells in the imaging field (Fig. 2D–F).

As the FAD^HI^ cells had a phenotype consistent with infiltrating macrophages, we next used F4/80, an antibody that marks most murine macrophages. We observed that over 75% of FAD^HI^ cells co-localized to the BV421-F4/80 positive cells (Fig. 2G–I). This result identifies the FAD^HI^ cells as predominantly macrophages. To confirm this result, we also used the macrophage selective marker, CD68, and found that many FAD^HI^ cells are also CD68 positive (Supplemental Figure).

It has been documented that macrophages phagocytose fluorescently labeled dextran^2^. Thus, we made use of labeled dextran to determine whether the FAD^HI^ population of cells was phagocytic. Texas red dextran injected into the tail vein was phagocytized by many of the FAD^HI^ cells (Supplemental Figure). These findings further confirm that the FAD^HI^ population represents macrophages.

### Fluorescence lifetime Imaging Microscopy of NADH shows that FAD^HI^ cells have a unique metabolic signature

**Figure 3:**
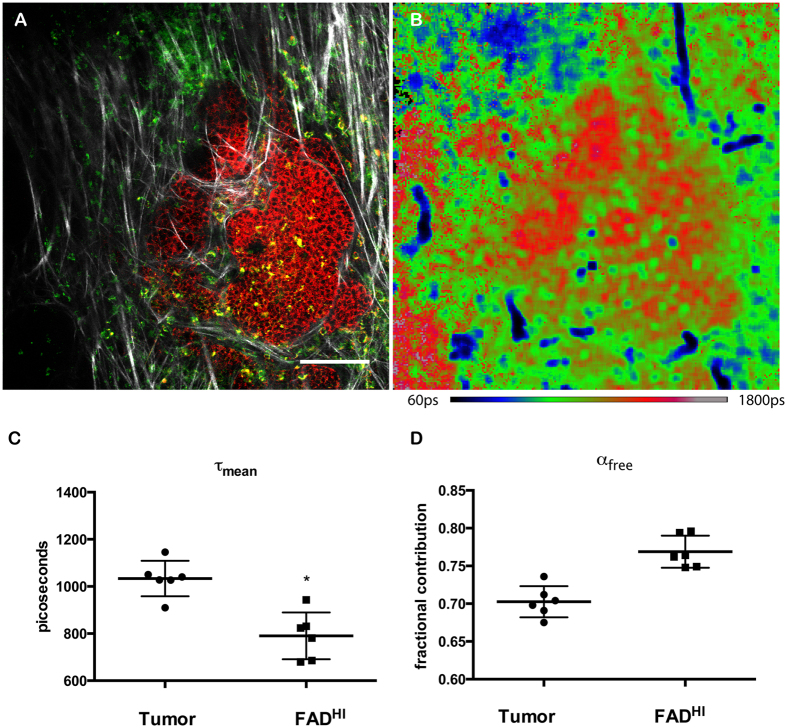
Fluorescence lifetime Imaging Microscopy of NADH shows that FAD bright cells have a more glycolytic metabolic signature. Endogenous Fluorescence of NADH showing tumor cells in breast microenvironment. (**A**) Composite of 3 channels; red = NADH^HI^, Green = FAD^HI^, Grayscale = SHG of collagen. Scale bar = 100 μm. (**B**) NADH-FLIM image of same field-of-view as in A. (**C**) Graph showing average lifetime comparing NADH^HI^ tumor cells to FAD^HI^ stromal cells. Data points are compiled from 6 regions of interest from 3 independent PyVT mice. Tumor NADH-FLIM values were determined from drawing a region of interest around the bright NADH tumor cells. FAD^HI^ region of interest was defined through thresholding FAD intensity to create ROI in FAD image and applying this to NADH FLIM image. The data point represents the average NADH FLIM value in this region of interest. Wilcoxon Rank Sum was performed to show statistical significance p = 0.02. (**D**) Graph showing the fractional contribution of the short lifetime component, free NADH, comparing NADH^HI^ tumor cells to FAD^HI^ stromal cells where p = 0.002.

NADH profiling by FLIM is useful as a means to determine changes in metabolic states. We next investigated whether the three distinct populations of cells that were identified via their endogenous fluorescence intensity of FAD and NADH also had differences in their respective NADH-FLIM signatures. Time Correlated Single Photon Counting (TCSPC) lifetime images were collected and the data fitted to a bi-exponential decay curve such that the short lifetime represents free NADH and the long lifetime represents bound NADH (Data not shown). An increase in the fraction of free NADH (α_FREE_) would correspond to a shorter weighted average fluorescence lifetime (τ_mean_). Data for the τ_mean_ of each pixel was color-mapped to provide spatial information related to the NADH-FLIM signature (Figure 3B). Using masks defined by FAD^HI^ and tumor cells defined by NADH^HI^ cells, NADH lifetime values were compared between FAD^HI^ stromal cells and NADH^HI^ tumor cells. Notably, the FAD^HI^ cells had a τ_mean_ that was significantly shorter than the tumor cells (Figure 3C), with a greater fractional component of free NADH (α_FREE_) (Fig. 3D). This suggests that these two cell types have distinct NADH lifetime characteristics that can be identified by NADH-FLIM.

### GFP-c-fms monocytic lineage cells have an NADH-FLIM signature that matches FAD^HI^ cells

**Figure 4:**
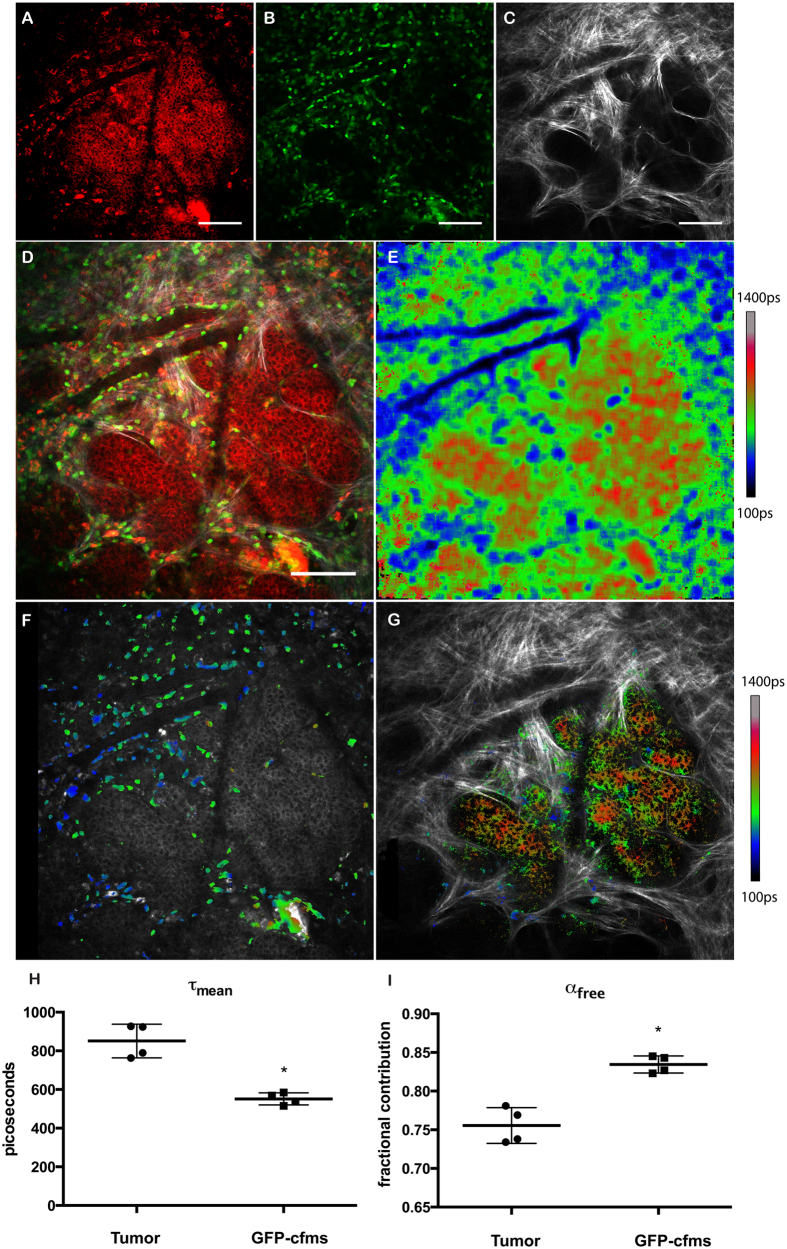
GFP- c-fms monocytic lineage cells have shorter NADH lifetimes than mammary tumor cells. (**A**) Endogenous Fluorescence of NADH^HI^ showing tumor cells in the breast microenvironment. (**B**) GFP c-fms cells in the breast tumor microenvironment. (**C**) SHG demonstrating collagen in the same field-of-view. Scale bar A-C = 100 μm. (**D**) Composite of the 3 channels shown in A-C: red = NADH^HI^, green = GFP-c*fms*, grayscale = SHG. Scale bar = 100 μm. (**E**) NADH-FLIM image of same field-of-view as in A-D. (**F**) NADH-FLIM image masked to show GFP-c*fms* positive cells overlayed on the NADH intensity image to provide comparison of stromal macrophages (heat map of NADH-FLIM) with NADH^HI^ tumor cells (grayscale). (**G**) NADH-FLIM image masked to show NADH^HI^ tumor cells overlayed on the SHG image to provide comparison of the tumor NADH-FLIM signature (heatmap) in the context of collagen structure (greyscale). (**H**) Graph showing average lifetime comparing GFP-c*fms* cells and tumor cells. Data points represent 4 regions of interest from 2 animals. Wilcoxon Rank sum was performed showing statistical significance with p = 0.03. (**I**) Graph showing the fractional contribution of the short lifetime component, free NADH, comparing GFP-c*fms* cells and tumor cells. Wilcoxon Rank sum was performed showing statistical significance with p = 0.03.

To further validate the use of NADH-FLIM as a means to identify macrophages, we crossed the MMTV-PyVT mice to mice bearing the GFP-*c-fms* transgene^27^,^28^,^29^. GFP-*c-fms* traces the monocytic lineage by expression of GFP driven by the monocyte-specific *c-fms* promoter. Because the excitation and emission of NADH (780 excitation 445/30 emission) differ from GFP (890nm excitation 520/35 emission) we were able to image NADH-FLIM in these lineage-traced monocytes (Fig. 4A–D). GFP-labeled monocytes showed an NADH-FLIM signature nearly identical to the FAD^HI^ cells, with a shorter τ_mean_ for NADH-FLIM than the tumor cells (Fig. 4H,I). These cells, like the FAD^HI^ cells, were located in the stroma and sparsely populated inside of the mammary tumor. These results demonstrate that the metabolic differences between monocytes and tumor cells can be exploited for imaging contrast.

By creating a monocyte-specific mask and a tumor-specific mask, we were able to spatially display the difference between NADH-FLIM in monocytic cells compared to tumor cells (Fig. 4E-G). Moreover, these images demonstrate metabolic heterogeneity in the tumor cell population.

## Discussion

It is becoming appreciated that tumor-associated macrophages play an important role in the progression of cancers. Using FLIM-based methods we find that the endogenous signature of FAD, NADH, and NADH-FLIM can be used to identify cell types *in vivo*, without the use of exogenous fluorophores. These unique cell profiles identify distinct populations of FAD^HI^ and NADH^HI^ cells. We propose that these represent distinct cell types. The PyVT tumor cells are represented by the NADH^HI^ signature. The notion that tumor cells have high NADH levels is consistent with their robust glycolytic nature, and the ability to label tumors by their ability to take up glucose, a property exploited for identification with fluoro-deoxyglucose in ^18^FDG-PET imaging. Multiple features are consistent with identifying the FAD^HI^ population as macrophages. These cells are highly phagocytic, consuming fluorescent dextran injected into the vasculature, and are stained by F4/80 and CD68, two antibodies selective, for macrophages. A limitation to this approach is that we still have a small set of FAD^HI^ cells that did not label with the F4/80 antibody, which could represent either another population, or that the antibody labeling is not totally robust. Moreover, the FAD^HI^ cells have a metabolic signature that matches the monocyte lineage, identified by expression of c-fms-GFP.

In addition to distinguishing cellular populations, the use of NADH-FLIM allows us to understand aspects of the metabolic state of these cells. Free NADH has a short lifetime (~0.44 ns) while protein-bound NADH has a long lifetime (~2.4 ns). We find that cytosolic NADH has a shorter τ_mean_ than does mitochondrial NADH (unpublished observations, and Bird *et al*.^17^). This is consistent with the idea that free NADH is representative of aerobic glycolysis in the cytosol, while bound NADH is more representative of oxidative phosphorylation in the mitochondria^16^,^30^. Moreover, we find a clear difference between the FAD^HI^ cells and NADH^HI^ cells in their metabolic profile determined by FLIM. FAD^HI^ cells have a significantly shorter NADH-FLIM τ_mean_ than the NADH^HI^ cells. These findings suggest that the FAD^HI^ macrophages have a more glycolytic-like signature than the tumor cells, consistent with observations that cells of monocytic linage exhibit more aerobic glycolysis then epithelial cells^31^. This is not to say that the tumor cells are not also exhibiting aerobic glycolysis, as suggested by Warburg^32^,^33^,^34^ as we see clear differences between tumor and normal breast cells *in vitro*^17^.

It must be noted, however, that the notion regarding a shorter τ_mean_ indicating a “more glycolytic” signature is likely an oversimplification, as there does not yet exist a complete understanding of the metabolic state as it relates to NADH-FLIM signatures. Future studies in which metabolomics are matched to FLIM signatures will further inform our understanding of the exact metabolic state that corresponds to different NADH-FLIM signatures. An additional nuance of these studies is that Nicotinamide Adenine Dinucleotide Phosphate (NADPH) is not spectrally distinguishable from NADH, and its contribution to the NADH-FLIM signature is not entirely clear. NADPH is a reactive mediator prominent in metabolic pathways such as Reactive Oxygen Species (ROS) and the Pentose Phosphate Pathway (PPP). Blacker *et al*. recently published evidence suggesting that NADPH presence and absence mostly effects the changes observed in the **τ**_**bound**_ component^22^. Notably, using their analytic approach, we do not see great to the value of the long lifetime component, consistent with a minimal contribution of NADPH to the FLIM signatures reported here.

FAD, NADH, and FLIM have been used to demonstrate the metabolic state of cells in several *in vitro* studies, and more recently to define redox states *in vivo*^16^,^35^,^36^. Here, we report for the first time to our knowledge the use of intravital, live, metabolic imaging *in vivo* to identify cell types in the tumor microenvironment. Because the tumor and stroma are largely undisturbed when imaging through a window, we are able to capture metabolic signatures without artifacts that may be introduced due to culture conditions, tissue excision, or fixation. These results highlight the unique potential that FLIM has in characterizing the tumor microenvironment non-invasively and quantitatively with high spatial and temporal resolution. With increased evidence that tumor-associated macrophages are involved in the progression of cancer, there is a great need for methods that can identify cell types and invasiveness of disease without the use of exogenous contrast. With an increase in interest in cellular metabolism in cancer and other diseases, FLIM also shows strong potential as a metabolic readout that can measure metabolic changes in cells with high spatial and temporal resolution. Various studies demonstrate changes in NADH-FLIM as it relates to progression of diseases in biopsied colon, breast and melanoma tissue^37^,^38^,^42^ and suggest that FLIM has a potential role in clinical diagnostics in the future. Further characterization of FLIM and its use in identifying metabolic changes and cellular contrast without any dyes could provide the groundwork to future tools that can aid clinicians and pathologists in rapid assessment of tumor features for advanced diagnostics of clinical tissue and models.

## Materials and Methods

### Animal Model and Mammary Imaging Window

All animal imaging and surgical protocols were approved by the Institutional Animal Use and Care Committee (IUCUC). All subsequent experiments were carried out in accordance to IUCUC guidelines. For all intravital experiments, the PyVT (MMTV-Polyoma Middle-T) animal model was used, which has been shown to model human breast cancer progression from premalignant to malignant tumor progression^39^. Tumors were palpable at 10 weeks. Observation of mammary tumors was done through surgical implementation of a Mammary Imaging Window (MIW)^24^. Windows were implanted at 11–13 weeks.

### Multiphoton Imaging Microscopy and Endogenous Fluorescence Imaging

All imaging was done at the Laboratory for Optical and Computational Instrumentation (LOCI) at the University of Wisconsin-Madison. Upright intensity Multiphoton Microscopy was conducted on the Ultima IV (Bruker Nano Surfaces, Middleton, WI^40^). Laser excitation on the Ultima IV was provided by the Insight (Spectra Physics, Palo Alto, CA). Detection was provided by Hamamatsu multi-alkali photomultiplier detectors. Data acquisition and scanning control was provided by PrairieView (Bruker Nano Surfaces, Middleton, WI). Images were gathered with Zeiss 20x 1.0 NA objective lens. NADH images were collected at 780 nm excitation and emission collected with 445/35 BP filter (Semrock). NADH^HI^ cells were defined as the upper 30% of pixel values in NADH image. FAD were collected at 890 nm excitation using 562/30 BP filter (Semrock). FAD ^HI^ cells were defined as the top 20% pixel intensities. All NADH^HI^ and FAD^HI^ images were subsequently thresholded using FIJI threshold and display only pixels in these regions.

### Fluorescent antibody *in vivo* imaging

By modifying the Mammary Imaging Window so that the end of a 30 gauge needle was located inside of the window, we were able to create a “ported” imaging window. This allows us the ability to inject fluorescent antibodies *in vivo* for identification of specific cells without disturbing the tumor microenvironment underneath the mammary imaging window. (See supplemental data). We used fluorescent antibodies that are conjugated to Brilliant Violet 421, as the emission spectrum can be readily separated from FAD. To prevent non-specific antibody binding, BD Fc Block (BD Bioscience) was injected into the window 20 minutes before BV421-antibody. Imaging was done using MPLSM at 780 nm. Emission was collected using 445/35 Bandpass filter (Semrock) for BV421 and 590/100 bandpass filter for FAD.

### Fluorescence Lifetime Imaging Microscopy (FLIM)

All FLIM images and their subsequent intensity images were collected with the Optical Workstation (LOCI, UW-Madison). Laser excitation was provided by Mai-Tai Deep See Ti:Sapphire laser (Spectra Physics, Palo Alto, CA) as previously described^41^. Data acquisition and scanning control was provided by WiscScan (LOCI, UW-Madison). Images were collected with 20x 1.0NA objective (Nikon) at 780 nm excitation. Time correlated single photon counting (TCSPC) was conducted on an SPC-830 photon counting board with DC-100 control electronics (Becker and Hickl, Berlin, Germany) and photon detection was conducted with Gallium Arsenide Phosphide photomultiplier tube H7422P-40 (GaAsP-PMT; Hamamatsu Photonics, Hamamatsu, Japan). FLIM data was fitted and analyzed using SPC Image (Becker and Hickl).

The average lifetime can then be written in the following equation:


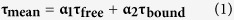


where τ_mean_ is the average lifetime, α_1_ is the fractional contribution of the τ_free_ component (short lifetime exponential), and α_2_ is the fractional contribution of τ_bound_ (long lifetime exponential) to τ_mean_. Thus a smaller τ_mean_ would have more free NADH component then a longer τ_mean_. Figure 4 lifetime overlay images were generated with the help of ImageJ Plugin Layers (Bob Dougherty, OptiNav).

### Statistics

The Wilcoxon Rank sum statistical test was performed to show significant differences between NADH τ_mean_ lifetime values and α_1_ to compare NADH^HI^ cells versus FAD^HI^ as well as NADH^HI^ versus GFP-cfms positive cells.

## Additional Information

**How to cite this article**: Szulczewski, J. M. *et al. In Vivo* Visualization of Stromal Macrophages via label-free FLIM-based metabolite imaging. *Sci. Rep.* **6**, 25086; doi: 10.1038/srep25086 (2016).

## Supplementary Material

Supplementary Information
