# Supplementary material for: In Vivo Visualization of Stromal Macrophages via label-free FLIM-based metabolite imaging
Source: Sci Rep. 2016 May 25;6:25086. doi: 10.1038/srep25086 (PMC4879594; doi:10.1038/srep25086)
Supplement: Supplementary file 1 [file srep25086-s1.doc]

Supplementary Data: ***In Vivo Visualization of Stromal Macrophages via label-free FLIM-based metabolite imaging***

Joseph M. Szulczewski, David R. Inman, David Entenberg, Julio Aguirre-Ghiso, James Castracane, John Condeelis, Kevin W. Eliceiri, Patricia J. Keely

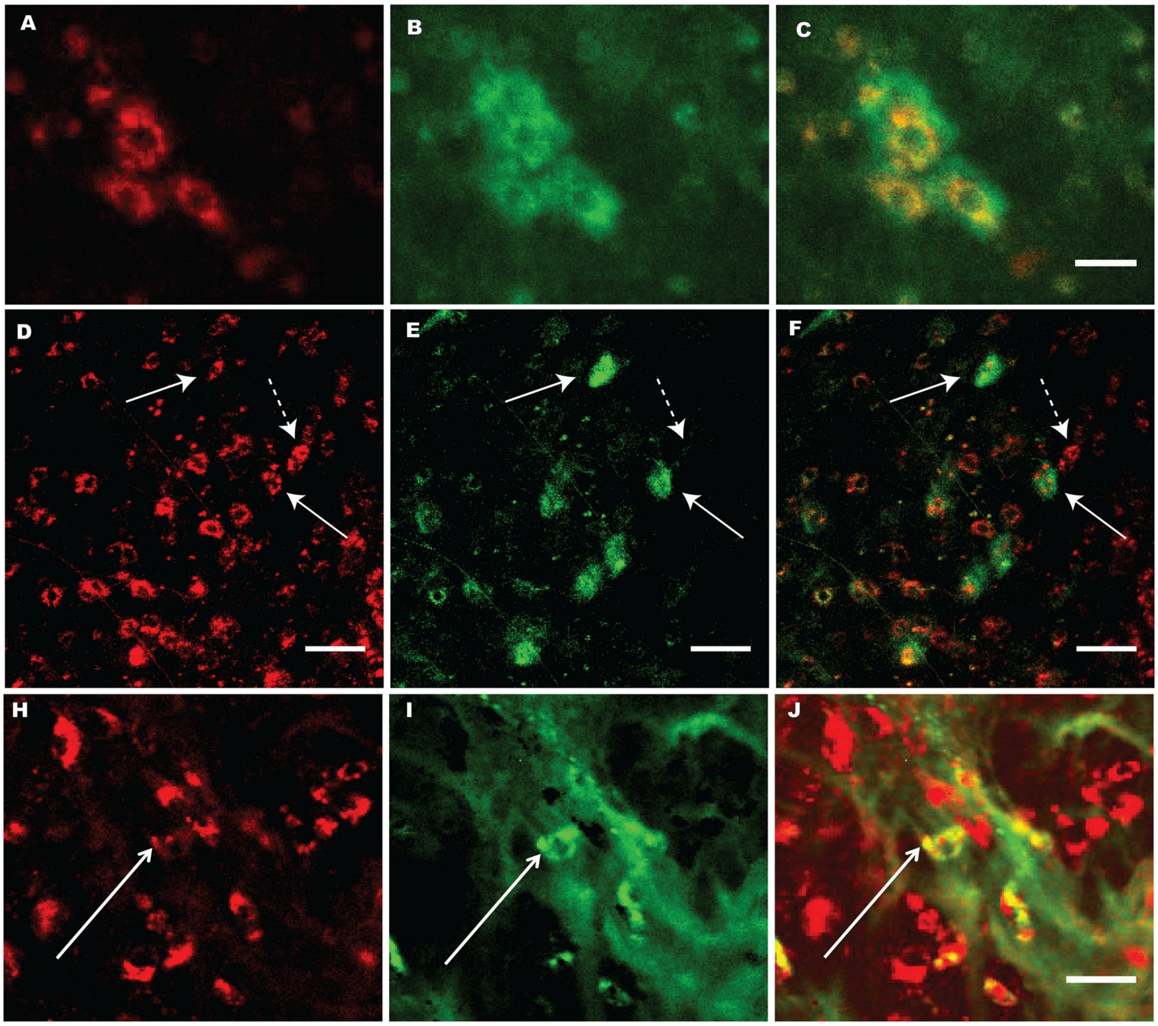

Supplemental Figure:
A-C Zoom in of F4/80 localization on FADHI cells. **A)** Zoom in field-of-view of FADHI cells in a PyVT tumor. **B)** Visualization of BV421-F4/80. **C)** Composite of M and N. Scale bar= 20µm.
D-F FADHI cells are also CD68+. Using BV421-CD68 primary conjugated antibody (Biolegend) co-localization of CD68 positive cells and FADHI cells were observed. Solid arrows show example of FADHI and CD68+ co localized cells. Dashed arrow shows FADHI cell only. **D)** FADHI cells in PyVT tumor. **E)** Visualization of of BV421-CD68 **F)** Composite of images from D and **E.** Scale bar= 50 µm.
H-J Phagocytosis of Texas-Red Dextran in FADHI cells. 200ul of Texas Red-Dextran (70kDa MW, 20mg/ml) was tail vein injected 2 hours before imaging in MMTV-PyVT mouse. FAD only cells were imaged at 780nm excitation with 590/100 BP filter. FAD and Texas Red Dextran were imaged at 890 with 590/100 BP filter. FAD intensity was subtracted from Texas Red Dextran + FAD image to produce Texas Red Dextran alone. **H)** FADHI cells in PyVT tumor. **I)** Texas-Red labeled Dextran image in PyMT tumor. **J)** Composite of FAD and Texas-red labeled Dextran showing co-localization of Texas Red dextran and FADHI cells (solid arrow). Scale Bar= 20 µm.
